# Malnutrition, cachexia and sarcopenia as susceptibility factors for cardiovascular complications in patients with cancer

**DOI:** 10.3389/fphar.2026.1762196

**Published:** 2026-02-13

**Authors:** Ionas Papasotiriou, Anastasios Tentolouris, Ioannis Ntanasis-Stathopoulos, Michalis Liontos

**Affiliations:** 1 Department of Clinical Therapeutics, School of Medicine, Alexandra General Hospital, National and Kapodistrian University of Athens, Athens, Greece; 2 First Department of Propaedeutic Internal Medicine and Diabetes Center, School of Medicine, Laiko General Hospital, National and Kapodistrian University of Athens, Athens, Greece

**Keywords:** cachexia, cancer, cardiotoxicity, malnutrition, sarcopenia

## Abstract

Cancer remains a leading cause of morbidity and mortality, although cancer survivorship has increased impressively in the past decades. Patients with cancer often face cardiovascular complications, either due to cancer itself or due to anti-cancer therapy, that may affect their quality of life and survival. Several studies have examined possible risk factors for cardiovascular susceptibility and/or cancer treatment-related cardiotoxicity, and some of them found a link between nutritional status and cardiovascular complications. In this review, we discuss the role of malnutrition, sarcopenia and cachexia, as susceptibility factors for cardiovascular complications and cardiotoxicity in cancer. The limited evidence shows that poor nutritional status and sarcopenia or cachexia is related to a cardiovascular burden in this population, and with a higher risk for cancer treatment-related cardiotoxicity. This relation may be mediated through several mechanisms and pathways, including cardiac wasting. Nutritional interventions should be examined for their effectiveness in reducing cardiovascular complications in patients with cancer, alongside with novel drugs that can prevent both skeletal and cardiac muscle wasting.

## Introduction

Cancer is a leading cause of morbidity and mortality worldwide, with incidence continuously increasing in most countries each year (Bray et al., 2024). Advances in disease screening, early diagnosis and treatment have led to higher survival rates, with many cancer patients exceeding 5-year or even 10-year survival (Wagle et al., 2025). However, many survivors subsequently experience comorbidities and late effects that can threaten longevity or significantly impair quality of life (Alshahrani et al., 2025; Zhao et al., 2025).

Cardiovascular complications are common among patients with malignancy. Cancer increases the risk of a broad spectrum of cardiovascular diseases through direct effects, shared risk factors, and the cardiotoxicity of cancer therapies (Strongman et al., 2019; Alshahrani et al., 2025). Cancer-related cardiovascular complications can reduce life expectancy, diminish quality of life through additional functional limitations, symptoms, and polypharmacy, and substantially increase healthcare costs for both patients and health systems (Strongman et al., 2019; Park et al., 2025). In some cases, they can even lead to premature death, with some patients dying from a cardiovascular event rather than from cancer itself (Alshahrani et al., 2025).

Nutritional status and body composition, especially muscle mass, have been identified as significant predictors of survival and other outcomes in cancer. Many studies have shown that malnutrition and sarcopenia are poor predictors of survival, disease progression and treatment response patients (Gascón-Ruiz et al., 2022; Park et al., 2025). Both conditions can occur by cancer itself, especially in malignancies located in the gastrointestinal track, since they affect appetite, absorption and ability of food intake (Cederholm et al., 2019). However, they are also common in other types of cancer, including solid and hematological tumors. Anti-cancer therapy, especially systemic therapy, often provokes symptoms like loss of appetite, vomiting, diarrhea and malabsorption, that can further affect nutritional status and muscle mass (Caillet et al., 2017; Arends et al., 2021; Powrózek et al., 2021).

Emerging evidence indicates that the presence of malnutrition, cachexia, or sarcopenia is associated with a higher risk of cancer treatment-related toxicities (Caillet et al., 2017; Roberto et al., 2024; Zhang Y. et al., 2024). Therefore, this review aims to provide a discussion on the limited evidence regarding malnutrition and sarcopenia as susceptibility factors for cardiovascular complications and treatment cardiotoxicity in patients with cancer.

## Cardiovascular burden of cancer

Cancer does not only affect the organ that is primarily located but additionally has an impact on many other organs and systems, often called paraneoplastic syndromes, including the heart (Pelosof and Gerber, 2010; Saha et al., 2025). It can lead to local and systemic metabolic changes, inflammation, oxidative stress, and immunological disturbances, all of which can affect the cardiovascular system (Saha et al., 2025). Inflammatory and pro-inflammatory molecules, especially cytokines, are elevated in many cancer types creating an inflammatory milieu. Cytokines like the tumour necrosis factor-a (TNF-a) and interleukins (IL-6, IL-1β) are produced by tumour cells, but also from other cells as response to cancer, leading to systemic inflammation (Candido and Hagemann, 2013; DeCotiis et al., 2016). The circulation of these cytokines affects the cardiovascular system through several pathways, since they promote atherogenesis, endothelial dysfunction and thrombosis (Pircher et al., 2012; Shang et al., 2023; Zhou et al., 2025). There is also evidence supporting that they participate in cardiomyocyte apoptosis and cardiac arrhythmia (Haudek et al., 2007; Wang et al., 2020; Zhu et al., 2022).

Tumour cells can also produce abnormal quantities of a protein called tissue factor (De Bono et al., 2023; Ren et al., 2023), which is responsible for blood clotting initiation, by binding to the factor VIIa and creating a complex that triggers the coagulation cascade (Zelaya et al., 2018). This mechanism has been considered responsible for cancer-associated thrombosis (Thomas et al., 2015). On the other hand, oxidative stress is another group of pathways that interlink cancer with cardiovascular complications. For example, reactive oxygens species (ROS), that are also overly produced in cancer (Szatrowski and Nathan, 1991), are involved in several processes including apoptosis and are an important contributor to cardiovascular burden though cardiomyocyte injury (Maalouf et al., 2012). Oxidative stress is also considered a major mechanism for treatment-related cardiotoxicity, especially in anthracycline-based therapies (McGowan et al., 2017).

Cancer and cardiovascular disease share overlapping risk factors (e.g., age, diabetes, obesity, smoking) and common pathophysiological mechanisms that further amplify cardiovascular risk (Alshahrani et al., 2025). Hence, cancer survivors continue to face a substantially increased likelihood of venous thromboembolism, heart failure or cardiomyopathy, arrhythmias, pericarditis, coronary artery disease, stroke, and valvular heart disease compared with the general population (Strongman et al., 2019). These risks peak during the first year following a cancer diagnosis but remain elevated for many years, with notable variation in magnitude and pattern across cancer types. Hematologic, lung, kidney, and ovarian cancers, in particular, have the highest risks of heart failure and thromboembolic events (Strongman et al., 2019).

The burden of cardiovascular disease among people with cancer varies substantially across studies, reflecting differences in cancer site and stage, age distribution, and whether analyses capture pre-existing versus incident cardiovascular disease. In a cross-sectional analysis of the National Health and Nutrition Examination Survey (NHANES 2015–2020) including 1,729 adults with a history of cancer, the weighted prevalence of CVD was 21.2% (Jiang et al., 2025). Also, prospective data from the ARIC (Atherosclerosis Risk in Communities) cohort, cancer survivorship has been linked to a substantially higher subsequent burden of incident cardiovascular disease (Florido et al., 2022). Among 12,414 participants (mean age 54 years; 55% female; 25% Black), 3,250 individuals developed incident cancer over a median 13.6 years of follow-up. Age-adjusted cardiovascular disease incidence rates were higher in cancer survivors than in those without cancer; 23.1 vs. 12.0 per 1,000 person-years. Notably, associations differed by cancer type, with breast, lung, colorectal, and hematologic or lymphatic cancers showing significant links to cardiovascular disease risk, unlike prostate cancer (Florido et al., 2022).

In a large, population-based retrospective cohort study of 4,519,243 adults and a median follow-up of 11.8 years, it was found that cancer was independently associated with higher risks of cardiovascular mortality, stroke, heart failure, and pulmonary embolism. This study also showed that cardiovascular risk was highest among patients with genitourinary, gastrointestinal, thoracic, nervous system, and hematologic malignancies, underscoring the need for coordinated cardio-oncology care across cancer sites (Paterson et al., 2022).

## Cancer-treatment related cardiotoxicity

Cancer therapies, including chemotherapy, targeted agents, radiotherapy and immunotherapy, have advanced remarkably over recent decades, transforming clinical outcomes. However, alongside these therapeutic gains, the cardiovascular burden of these therapies constitutes a major clinical concern, also called as cancer therapy-related cardiovascular toxicity (Lyon et al., 2022; Bloom et al., 2025). These toxicities may present acutely during treatment or arise as late complications, sometimes manifesting years after therapy has been completed. Clinically, they have a broad range of conditions, such as heart failure, cardiomyopathy, arrhythmias, hypertension, myocarditis, pericardial disease, coronary artery disease, valvular dysfunction and embolism (Lyon et al., 2022; Tetterton-Kellner et al., 2024).

Anthracyclines such as doxorubicin and daunorubicin are among the most well-established agents linked to cardiotoxicity, carrying a dose-dependent risk of heart failure due to direct myocardial injury (Henriksen, 2018). Human epidermal growth factor receptor 2 (HER2) - targeted agents, such as trastuzumab, are essential in breast cancer treatment but can cause typically reversible heart failure, particularly when administered with anthracyclines (Yang et al., 2021). In contrast to anthracycline-induced cardiotoxicity, which is characteristically dose-dependent and often irreversible, trastuzumab-related heart failure generally improve following treatment interruption and initiation of directed heart failure therapy (Yang et al., 2021). In a systematic review and meta-analysis by Guenancia et al. (2016) it was found that obesity was a significant risk factor for developing cardiotoxicity in breast cancer patients receiving anthracyclines, with or without trastuzumab (Guenancia et al., 2016).

Alkylating agents such as cyclophosphamide can also lead to cardiotoxicity, most commonly presenting acutely within 1–10 days of high-dose administration (Iqubal et al., 2019). Other common anti-cancer drugs with evidence for a possible cardiotoxic effect include carfilzomib, tyrosine kinase inhibitors (TKIs), immune checkpoint inhibitors (ICI), and some monoclonal antibodies among others (Lyon et al., 2022; Papassotiriou et al., 2025; Tentolouris et al., 2025).

Apart from chemotherapy, radiation therapy involving the thoracic region can also induce long-term cardiotoxicity through direct injury to virtually all cardiac structures, including the pericardium, myocardium, coronary arteries, valves, and the conduction system. Radiation-induced heart disease is often insidious, with clinical manifestations emerging from years to decades after exposure (Zhao et al., 2025).

As cancer survivorship continues to rise and patients live longer, the early recognition, systematic surveillance, and proactive management of cardiotoxicity have become essential components of modern oncologic care (Lyon et al., 2022). The optimal moment to initiate cardiovascular prevention in patients with cancer is at the time of diagnosis and before the onset of cancer therapy. This approach enables the oncology team to account for CV risk when selecting treatment regimens, to educate patients regarding their individualized risk profile, to tailor surveillance and follow-up strategies, and to refer high-risk individuals to cardio-oncology services when appropriate (Lyon et al., 2022).

## Cachexia, sarcopenia and cardiac wasting in cancer

Although cancer cachexia and sarcopenia are considered two distinct conditions (Arends et al., 2021), in this context they are examined together in the common basis of muscle wasting, due to limited evidence. Cancer can lead to both muscle and cardiac cachexia, two conditions characterized by muscle and cardiac muscle wasting, and in some cases even atrophy (Rausch et al., 2021). However, cancer cachexia expands beyond weight and muscle loss and is considered by many scientific groups as a syndrome where muscle loss is accompanied by inflammation, anorexia and other metabolic disturbances (Mattox, 2017; Arends et al., 2021; Soria Rivas et al., 2024). On the other hand, sarcopenia is similar to the dimension of muscle loss in cancer cachexia but is also linked to functional loss, such as loss of muscle strength (Papadopoulos et al., 2025). Although both conditions present an overlapping regarding their phenotypic features, cachexia is more relevant to disease progression, while sarcopenia is more prevalent among older cancer patients (Tinsley-Vance et al., 2024; Papadopoulos et al., 2025). Both conditions are common in cancer. A wide meta-analysis with data from 81,814 cancer patients showed that the prevalence of sarcopenia in a mixed solid tumor sample was 35%, with a higher prevalence (>50%) among patients with esophageal, urothelial, prostate and other cancers (Surov and Wienke, 2022). The prevalence of cancer cachexia may also differ among different cancer populations, where a frequency of over 50% has been reported among older adults (Poisson et al., 2021). However, cancer cachexia can also exist even without sarcopenia. A large prospective study among 1215 patients with gastric cancer showed that cancer cachexia was present in 27% of patients, while sarcopenia in 20% of them. This study also showed different prognostic values between these two conditions, with sarcopenia being more significant among early stage older patients, and cachexia more significant in advanced stages of the disease (Zhuang et al., 2022). Despite their different clinical features, the common atrophic and muscle wasting basis of both conditions is linked with cardiac consequences.

Cancer-related cardiac wasting and atrophy have been found in both animal models and in humans, including autopsy evidence in patients with cancer (Anker et al., 2024). Cardiac wasting, although disease driven, have been also found to be related with weight loss in these patients (Barkhudaryan et al., 2017). There is evidence that cardiac remodeling and wasting in cancer, occurs even without cachexia (Barkhudaryan et al., 2017; Lena et al., 2023). However, co-existence of cachexia seems to intensify this phenomenon (Lena et al., 2023).

On molecular basis, several mechanisms have been proposed for cancer cachexia and muscle mass loss, that could also link the association between sarcopenia and/or cachexia with cardiac wasting in cancer. The chronic systemic inflammation, often observed in cancer, is characterized by the release of potentially cachectic molecules, such as leptin, TNF-a and other cytokines (Mantovani et al., 2000; Porporato, 2016). These molecules can affect brain activity and promote appetite reduction and hormonal changes linked to muscle catabolism (Vudatha et al., 2022). In addition, vascular dysfunction, through the activin A–PGC1α axis, has been also suggested as another possible mechanism of cancer cachexia (Muscaritoli et al., 2017).

Bas et al. (2023) found an association between sarcopenia and anthracycline cardiotoxicity, with patients with sarcopenia having more than 3 times the risk for developing cardiotoxicity. In this study, sarcopenia was assessed using computed tomography scans (Bas et al., 2023). However, a limitation of the study is that sarcopenia was assessed using different indices across patients. Specifically, the skeletal muscle index or the psoas muscle index were used. In another study of a mixed population of patients with cancer, it was found that the pectoralis muscle mass index was a significant predictor of cardiotoxicity related to anthracyclines, where patients with higher values of this index had lower risk for major adverse cardiac events (Toama et al., 2022).

## Malnutrition and cardiovascular complication in cancer

Malnutrition is a state of reduced food intake, that can lead to body weight and muscle mass loss. According to the Global Leadership Initiative on Malnutrition (GLIM) criteria, malnutrition is defined by a positive malnutrition screening test, and at least one phenotypic criterion (unintentional weight loss, low body mass index or reduced muscle mass) and one etiologic (reduced food intake or inflammation/disease burden) criterion (Cederholm et al., 2019).

Although malnutrition is often accompanied by sarcopenia, low muscle mass may not be present at its initial stages but still predisposes patients to worse outcomes. There are many data indicating that malnourished patients with cancer are at higher risk for mortality, disease progression, adverse events and dose-limiting toxicities (Cederholm et al., 2019). In a prospective study were the GLIM criteria for malnutrition were used, it was shown that malnourished patients with cancer were at higher risk for cancer treatment toxicities; however, cardiovascular toxicities were not examined in this study (Gascón-Ruiz et al., 2022). As reported for sarcopenia and cachexia, systemic inflammation has been also reported in malnutrition (Muscaritoli et al., 2017), linking the pathophysiological basis for a cardiotoxic environment. The cardiac burden of malnutrition is also supported by evidence on non-oncologic patients. For example, higher levels of the N-terminal pro-brain natriuretic peptide were found in malnourished patients with idiopathic pulmonary hypertension (Zhang S. et al., 2024).

## Methodological issues

Studies on the role of malnutrition, cachexia and sarcopenia on cardiovascular complication and cardiotoxicity in cancer are scarce, and the available studies are presented with high heterogeneity on several key methodological points. Despite the need for more studies on this issue, it is necessary to have studies using widely accepted criteria for each condition. After the publication of the GLIM criteria for malnutrition (Cederholm et al., 2019), most studies comply with the use of these criteria to define malnutrition, and this should be also incorporated into studies examining the relation between malnutrition and cancer related cardiotoxicity. The use of the terms sarcopenia and cachexia is likewise inconsistent across the literature. Studies referring to sarcopenia may actually refer to cachexia, since the latter is more disease-specific in patients with cancer (Arends et al., 2021). However, this confusion is reasonable, since sarcopenia may exist without cachexia, while cachexia rarely exists without sarcopenia and in patients with cancer, characteristics other than low muscle mass are often present (Porporato, 2016; Arends et al., 2021).

Another methodological issue observed in the current literature is the marked heterogeneity in the definitions used for cancer-related cardiovascular complications. Various studies employ differing diagnostic criteria, timeframes, and clinical parameters, resulting in inconsistent classifications and making direct comparisons between findings challenging. This variability complicates the interpretation of outcomes and highlights the need for standardised definitions to improve the reliability and reproducibility of research in this field.

In addition, variation in the methodologies used for the diagnosis of sarcopenia presents a significant challenge in both clinical and research contexts. For instance, certain studies employ computed tomography (CT) imaging to quantify muscle mass, frequently measuring parameters such as the skeletal muscle index or the psoas muscle index, whereas others may rely on the pectoralis muscle mass index or alternative radiological modalities. The absence of a unified diagnostic approach can lead to considerable discrepancies in the identification of sarcopenia across cohorts, thereby complicating direct comparisons of findings between studies. Furthermore, the adoption of divergent measurement sites, cut-off thresholds, and definitional criteria further contributes to variability in the reported prevalence and the observed clinical associations of sarcopenia among individuals with cancer. This pronounced heterogeneity in diagnostic criteria and assessment tools underscores the critical need for consensus and standardization, which would enable a more accurate evaluation of the relationship between sarcopenia and cardiovascular complications, as well as treatment outcomes, in oncology populations.

## Future directions

It is of great significance to explore whether the management of malnutrition and sarcopenia or cachexia can reduce the cardiovascular burden among cancer patients, especially those under cardiotoxic treatments. Given the rapidly expanding population of long-term cancer survivors and the increasing recognition of cardiovascular morbidity as a major determinant of outcomes, there is an urgent need to move beyond association studies and define preventive, scalable care pathways. In this direction, it is also important to examine possible treatments for malnutrition and sarcopenia. In a recent pre-clinical study by Quagliariello et al. (2024) it was found that vericiguat, a new guanylate cyclase activator used for heart failure, may exert both cardioprotective and anti-sarcopenic effects (Quagliariello et al., 2024). Studies in this direction may open a new era in the management of both sarcopenia or cachexia and cancer treatment related cardiotoxicity, alongside with nutritional interventions. Although there is adequate literature about the effectiveness of nutritional and pharmaceutical interventions on malnutrition and sarcopenia or cachexia in cancer patients, it is important to examine whether such interventions also translate into cardioprotective benefits.

Priorities for future research include prospective, therapy-specific cardio-oncology cohorts that enroll patients at diagnosis or before treatment initiation and apply standardized definitions of malnutrition and sarcopenia/cachexia (including objective body composition measures) alongside systematic cardiovascular phenotyping. Such studies should capture adjudicated cardiovascular outcomes, such as heart failure, arrhythmias, thromboembolism, stroke, and CV mortality, and incorporate biomarkers and imaging to clarify mechanistic links between muscle wasting, inflammation, and cardiac vulnerability. There is also need for interventional trials to test multimodal strategies, nutritional support, therapeutic exercise and selected pharmacologic approaches in high-risk populations, with cardiovascular outcomes as primary or secondary endpoints.

Even though cardiovascular programmes and guidelines have been issued for the monitoring and management of individuals with cardiovascular-related complications, their implementation in populations affected by malnutrition remains particularly important, given that malnutrition may be asymptomatic (Simões et al., 2018).
